# Training Image Free High-Order Stochastic Simulation Based on Aggregated Kernel Statistics

**DOI:** 10.1007/s11004-021-09923-3

**Published:** 2021-02-12

**Authors:** Lingqing Yao, Roussos Dimitrakopoulos, Michel Gamache

**Affiliations:** 1 Department of Mathematics and Industrial Engineering, Polytechnique Montréal, Montreal, QC H3T 1J4 Canada; 2 COSMO–Stochastic Mine Planning Laboratory, Department of Mining and Materials Engineering, McGill University, 3450 University Street, Montreal, QC H3A 2A7 Canada

**Keywords:** High-order sequential simulation, Statistical learning, Spatial statistics, Kernel space

## Abstract

A training image free, high-order sequential simulation method is proposed herein, which is based on the efficient inference of high-order spatial statistics from the available sample data. A statistical learning framework in kernel space is adopted to develop the proposed simulation method. Specifically, a new concept of aggregated kernel statistics is proposed to enable sparse data learning. The conditioning data in the proposed high-order sequential simulation method appear as data events corresponding to the attribute values associated with the so-called spatial templates of various geometric configurations. The replicates of the data events act as the training data in the learning framework for inference of the conditional probability distribution and generation of simulated values. These replicates are mapped into spatial Legendre moment kernel spaces, and the kernel statistics are computed thereafter, encapsulating the high-order spatial statistics from the available data. To utilize the incomplete information from the replicates, which partially match the spatial template of a given data event, the aggregated kernel statistics combine the ensemble of the elements in different kernel subspaces for statistical inference, embedding the high-order spatial statistics of the replicates associated with various spatial templates into the same kernel subspace. The aggregated kernel statistics are incorporated into a learning algorithm to obtain the target probability distribution in the underlying random field, while preserving in the simulations the high-order spatial statistics from the available data. The proposed method is tested using a synthetic dataset, showing the reproduction of the high-order spatial statistics of the sample data. The comparison with the corresponding high-order simulation method using TIs emphasizes the generalization capacity of the proposed method for sparse data learning.

## Introduction

Stochastic simulation methods are used to quantify the uncertainty of spatially distributed attributes of geological and other natural phenomena. It is well known that the conventional second-order stochastic simulation methods are limited in reproducing the complex patterns or nonlinear features exhibited in the spatial attributes of interest (Journel and Deutsch 1993; Xu 1996; Journel 2005). The so-called multiple point simulation (MPS) methods (Guardiano and Srivastava 1993; Strébelle 2000, 2002; Journel 2003; Arpat 2005; Zhang et al. 2006; Wu et al. 2008; Remy et al. 2009; Mariethoz et al. 2010; Mariethoz and Caers 2014) have been developed to address the limitation of conventional simulation methods based on the concept of multiple-point statistics. The multiple-point simulation framework introduced training images (TI) as statistical analogs of the spatial attributes under consideration. The multiple-point statistics are either (a) captured by occurrences of data events formed by indicators at multiple locations inside the so-called spatial templates when the spatial attributes are categorical, or (b) generalized to continuous data as the pattern similarity among patches from the TI and the proceeding simulation. The multiple-point statistics described in the MPS methods are based on a certain spatial template, however, are limited given that they do not consistently consider the lower-order spatial statistics in the related sub-templates. In addition, although the utilization of a TI as prior information to account for multi-point interactions of spatial attributes is conceptually appealing and justified (Journel 2003), generally, the information from TI is not conditioned to the available data. Thus, the potential statistical conflicts existing between the sample data and the TI is a hindrance for the TI-driven MPS methods to reproduce the spatial patterns properly. This issue seems more prominent when the sample data are relatively dense, as in mining applications (Goodfellow et al. 2012). Improvements of the MPS realizations may be possible by either transforming the original TI to increase its consistency to the actual data (Straubhaar et al. 2019), or by explicitly imposing constraints on the realizations to ameliorate the potential conflicts of the simulation and the TI (Shahraeeni 2019). However, these improvements do not change the TI-driven nature of the MPS methods.

The high-order simulation methods provide a new framework to simulate complex spatial patterns, addressing the drawbacks in MPS methods as discussed in the related publications (Dimitrakopoulos et al. 2010; Mustapha and Dimitrakopoulos 2010a, 2011; Minniakhmetov and Dimitrakopoulos 2016, 2017; Minniakhmetov et al. 2018; Yao et al. 2018, 2020; de Carvalho et al. 2019). The high-order simulation methods equip the multiple-point spatial structures with well-defined mathematical entities, such as spatial cumulants or high-order spatial moments (Dimitrakopoulos et al. 2010; Mustapha and Dimitrakopoulos 2010b; Minniakhmetov et al. 2018). Related program for computing spatial cumulants is available (Mustapha and Dimitrakopoulos 2010a), and its computational efficiency can be further improved by parallelization using GPU (Li et al. 2014). The random field model in the high-order simulation framework makes no assumption on any specific probability distribution. Instead, a Legendre polynomial expansion series is adopted to approximate the underlying distribution, where spatial cumulants are quantified to infer the expansion coefficients (Mustapha and Dimitrakopoulos 2010a, 2011). To cope with the statistical conflicts between the samples and the TI, the high-order simulation methods take into account both the high-order spatial statistics from the sample data and the TI. However, the latter ones are only incorporated when the replicates from the sample data are insufficient for inference and, therefore, limit the influence of the TI on the realizations (Mustapha and Dimitrakopoulos 2010a, 2011). Minniakhmetov and Dimitrakopoulos (2017) propose a high-order simulation method without TI, which uses instead special relations of high-order indicator moments in boundary conditions related to a certain spatial template. However, these mathematical relations can only be established for categorical random variables. Yao et al. (2020) propose a statistical learning framework of high-order simulation in kernel space by constructing a so-called spatial Legendre moment kernel from a new computational model of high-order simulation based on spatial Legendre moments (Yao et al. 2018). The proposed statistical learning framework in Yao et al. (2020) demonstrates the advantage of its generalization capacity with regards to improving of the numerical stability, as compared with the previous high-order simulation methods. This generalization capacity also mitigates the statistical conflicts between the samples and the TI. This is due to the fact that the high-order spatial statistics are adjusted to the target probability distribution through the learning process, as opposed to directly being incorporated into the coefficients of polynomial expansion series as with the other methods. The simulation under a statistical learning framework (Yao et al. 2020) proceeds sequentially according to a random path based on the sequential decomposition of the multivariate distribution of the random field model (Rosenblatt 1952; Journel 1994). Specifically, the replicates are mapped onto the spatial Legendre moment space and the empirical kernel statistics are computed thereafter. The target probability distributions are also embedded into the same kernel space to obtain the expected kernel statistics. Matching these two elements in the kernel space leads to a minimization problem in the quadratic form determined by the kernel function. Solving the minimization problem leads to target probability distributions that comply with the high-order spatial statistics of the available data.

The present paper proposes fundamental adjustments of the above statistical learning framework so that it becomes more suitable for sparse data learning, thus allowing the development of a TI-free high-order simulation method for the continuous spatial attributes. The motivation of this development is to utilize the more reliable sample data for inference of high-order spatial statistics and avoid the potential conflicts from using the TI, while addressing the issue of data sparsity. Since retrieving replicates that fully match the spatial template of the data events is difficult due to the sparsity of the sample data, it is worth noting that replicates that are partially matched to the spatial template may exist. These partially matched replicates, nevertheless, provide useful and relevant information to the related statistical inference, while determining how to utilize this incomplete information remains a challenge. The above-mentioned matters are addressed herein by a proposed concept of aggregated kernel statistics. More specifically, each spatial template is associated with a certain kernel subspace, such that any replicate associated with the same spatial template can be mapped onto an element of the corresponding kernel space. Accordingly, these mapped elements in the kernel subspaces are utilized to compute the kernel statistics. The kernel statistics in a set of kernel subspaces are combined to determine the aggregated kernel statistics through the relations introduced in this paper. Eventually, the aggregated kernel statistics are embedded into the kernel subspace corresponding to the conditional probability distribution encountered in the high-order sequential simulation framework, and the statistical learning algorithm is applied to approximate a conditional probability distribution.

The remainder of the paper is organized as follows. Firstly, the mathematical concepts and the proposed method are presented. Next, a case study from a synthetic dataset is used to assess the performance of the proposed method and demonstrate its practical aspects. Conclusions follow.

## Method

Consider the spatial attributes of interest distributed on a discrete grid as a random field model denoted by ${\varvec{Z}}\left( {\varvec{u}} \right)$ with ${\varvec{u}} = \left\{ {{\varvec{u}}_{1} ,{\varvec{u}}_{2} , \ldots ,{\varvec{u}}_{n} } \right\}$ corresponding to various locations within the grid, then ${\varvec{Z}}\left( {\varvec{u}} \right) = \left\{ {Z\left( {{\varvec{u}}_{1} } \right),Z\left( {{\varvec{u}}_{2} } \right), \ldots ,Z\left( {{\varvec{u}}_{n} } \right)} \right\}$ comprises a multivariate probability distribution $f_{{\varvec{Z}}}$ given that $Z\left( {{\varvec{u}}_{i} } \right)$ representing random variables at location ${\varvec{u}}_{i} \user2{ }\left( {i = 1, \ldots ,n} \right)$. Under the sequential simulation framework (Journel 1994), the joint probability distribution $f_{{\varvec{Z}}}$ is decomposed into a sequence of conditional probability distributions following a random path to visit the entire simulation grid, and random values are drawn from these conditional probability distributions sequentially along the random path to generate one realization. Both the available sample data and the previous simulated attribute values are considered as the conditioning data throughout the simulation process.

Without loss of generality, suppose that the current attribute $Z\left( {{\varvec{u}}_{0} } \right)$ to be simulated locates at ${\varvec{u}}_{0}$, and the informed data $\left\{ {\zeta_{1} , \ldots ,\zeta_{N} } \right\}$ at the surrounding locations ${\varvec{u}}_{0} + {\varvec{h}}_{1} , \ldots , {\varvec{u}}_{0} + {\varvec{h}}_{N}$, consist of a data event as the conditioning data. From the geometric configuration of the data event, a spatial template ${\varvec{T}} = \left\{ {{\varvec{u}}_{0} ,{\varvec{u}}_{0} + {\varvec{h}}_{1} , \ldots , {\varvec{u}}_{0} + {\varvec{h}}_{N} } \right\}$ can be determined with the distance vectors ${\varvec{h}}_{1} , \ldots ,{\varvec{h}}_{N}$ pointing outwards from the center ${\varvec{u}}_{0}$ to the surrounding locations. Let the conditional probability density function (CPDF) be denoted as $f(z_{0} |\zeta_{1} , \ldots ,\zeta_{N} )$. The key task to derive the CPDF is achieved by a statistical learning algorithm in kernel space herein. The related replicates associated with template ${\varvec{T}}$ are retrieved from the sample data, and these replicates are used as the training data of statistical learning to infer the underlying probability distribution. Specifically, the retrieved replicates are mapped to elements in kernel spaces to build kernel statistics carrying the high-order spatial information from the replicates. The aggregated kernel statistics are proposed, allowing to incorporate the high-order spatial statistics from the ensemble of replicates with different spatial configurations. The target CPDF is then achieved by the statistical learning algorithm approaching the aggregated kernel statistics from the sample data.

### Aggregation of Spatial Legendre Kernel Subspaces and Kernel Statistics

#### Spatial Legendre Moment Kernel Subspaces

The kernel space is a Hilbert space defined through a positive definite kernel function. Legendre polynomials are orthogonal polynomials defined on interval [−1, 1]. The Legendre polynomial expansion series can approximate arbitrary piecewise continuous function and are used for approximation of probability density function in high-order simulations (Mustapha and Dimitrakopoulos 2010a). The spatial Legendre moment reproducing kernel (SLM-kernel) (Yao et al. 2020) is derived from a new computational model for high-order simulation (Yao et al. 2018) based on the Legendre polynomial series. The SLM-kernel carries the information of high-order spatial statistics so that the density estimation in the high-order sequential simulation could be achieved by a statistical learning process in kernel space. The SLM-kernel can be defined to associate a kernel subspace to random variables within a certain spatial template. Given a set of random variables $V = \left\{ {Z_{0} ,Z_{1} , \ldots ,Z_{N} } \right\}$ with nodes corresponding to spatial template ${\varvec{T}} = \left\{ {{\varvec{u}}_{0} ,{\varvec{u}}_{0} + {\varvec{h}}_{1} , \ldots , {\varvec{u}}_{0} + {\varvec{h}}_{N} } \right\}$, the kernel subspace can be determined by a spatial Legendre moment reproducing kernel (SLM-kernel) as

1 $$ K_{V} \left( {{\varvec{Z}},\user2{Z}^{\prime}} \right) = \mathop \prod \limits_{i = 0}^{N} \left[ {\mathop \sum \limits_{w = 0}^{W} \left( {w + \frac{1}{2}} \right)P_{w} \left( {z_{i} } \right)P_{w} \left( {z_{i}^{\prime} } \right)} \right], $$

where $N$ corresponds to size of the spatial template. ${\varvec{Z}} = \left( {z_{0} ,z_{1} , \ldots ,z_{N} } \right), \user2{Z}^{\prime} = \left( {z_{0}^{\prime} ,z_{i}^{\prime} , \ldots ,z_{N}^{\prime} } \right)$ are attribute values corresponding to spatial template ${\varvec{T}}$ and $P_{w} \left( \cdot \right)$ is the Legendre polynomial of order $w$ and $W$ is the maximal order of Legendre polynomials under consideration. Let the original data space denote as ${\mathbb{E}}$ and the kernel space associated to kernel $K$ denote as ${\mathcal{H}}$, the canonical feature map (Steinwart and Christmann 2008), $\phi \left( t \right):{\mathbb{E}} \to {\mathcal{H}},t \mapsto K\left( {., t} \right),\forall t \in {\mathbb{E}}$, defines a valid feature map that takes an element from the original data space to an element in the kernel subspace. In other words, after the feature mapping, each element in the original data space ${\mathbb{E}}$ has a “representer” in the kernel space ${\mathcal{H}}$.

#### Aggregated SLM-Kernel Statistics

If a training image (TI) is provided as an exhaustive dataset, most of the replicates of a data event fully match the spatial configuration of the data event while the partially matched ones are negligible. The replicates of a data event from the sample data, however, include both fully matched and partially matched replicates that correspond to different configuration of spatial templates. Therefore, the replicates are respectively mapped to different kernel subspaces. Kernel statistics, in general, means either the empirical statistics from the mapped elements or the expected statistics in the kernel subspaces, such as empirical mean and expectation. Equation (1) suggests that replicates associated with different spatial templates would be mapped to kernel subspaces with different kernel functions. The kernel statistics associated with different spatial templates thus come from different subspaces and need to be combined appropriately to get the aggregated kernel statistics for the inferring of underlying probability distribution afterwards.

For convenience, the following notation is defined to clarify the relations between the spatial templates. Given a template ${\varvec{T}} = \left\{ {{\varvec{u}}_{0} ,{\varvec{u}}_{0} + {\varvec{h}}_{1} , \ldots , {\varvec{u}}_{0} + {\varvec{h}}_{N} } \right\}$ as a set of locations with the center node denoted as ${\rm center}\left( {\varvec{T}} \right) = {\varvec{u}}_{0}$, the size of the ${\varvec{T}}$ is the same as the number of the elements in it and is denoted as $\left| {\varvec{T}} \right|$, i.e., $\left| {\varvec{T}} \right| = N + 1$ here. Since the replicates of the data events are matched by their relative positions to the center node regardless of the location of the center node, the relations between the spatial templates are defined in the same manner. Let ${\varvec{T}}_{a} = \left\{ {{\varvec{u}}_{a} ,{\varvec{u}}_{a} + {\varvec{h}}_{1} , \ldots ,{\varvec{u}}_{a} + {\varvec{h}}_{{N_{a} }} } \right\}$ and ${\varvec{T}}_{b} = \left\{ {{\varvec{u}}_{b} ,{\varvec{u}}_{b} + {\varvec{h}}_{1} , \ldots ,{\varvec{u}}_{b} + {\varvec{h}}_{{N_{b} }} } \right\}$ be the two spatial templates under consideration, then the relations between ${\varvec{T}}_{a}$ and ${\varvec{T}}_{b}$ are the following:

1. If $\left| {{\varvec{T}}_{a} } \right| = \left| {{\varvec{T}}_{b} } \right|$, $\forall t_{a} \in {\varvec{T}}_{a}$, $\exists !t_{b} \in {\varvec{T}}_{b}$, such that $t_{a} - {\rm center}\left( {{\varvec{T}}_{a} } \right) = t_{b} - {\rm center}\left( {{\varvec{T}}_{b} } \right)$, then ${\varvec{T}}_{a}$ and ${\varvec{T}}_{b}$ have the same geometry configuration and the identical relation is expressed as ${\varvec{T}}_{a} = {\varvec{T}}_{b}$.
2. If $\left| {{\varvec{T}}_{a} } \right| \le \left| {{\varvec{T}}_{b} } \right|$, $\forall t_{a} \in {\varvec{T}}_{a}$, $\exists !t_{b} \in {\varvec{T}}_{b}$, such that $t_{a} - {\rm center}\left( {{\varvec{T}}_{a} } \right) = t_{b} - {\rm center}\left( {{\varvec{T}}_{b} } \right)$, then ${\varvec{T}}_{b}$ contains the geometry configuration as a subset and the relation is expressed as ${\varvec{T}}_{a} \subseteq {\varvec{T}}_{b}$ or ${\varvec{T}}_{b} \supseteq {\varvec{T}}_{a}$. If $\left| {{\varvec{T}}_{a} } \right| < \left| {{\varvec{T}}_{b} } \right|$ strictly, the above relation is expressed as ${\varvec{T}}_{a} \subset {\varvec{T}}_{b}$ or ${\varvec{T}}_{b} \supset {\varvec{T}}_{a}$.

Suppose that the spatial template of the conditioning data is ${\varvec{T}} = \left\{ {{\varvec{u}}_{0} ,{\varvec{u}}_{0} + {\varvec{h}}_{1} , \ldots , {\varvec{u}}_{0} + {\varvec{h}}_{N} } \right\}$ and that the nodes are ordered increasingly according to their distances from the center. By dropping the furthest node from the template ${\varvec{T}}$ each time, a hierarchical set of spatial templates can be defined as

2 $$ v_{N} = {\varvec{T}} \supseteq \user2{ }v_{N - 1} = \user2{ T}\backslash \left\{ {{\varvec{u}}_{0} + {\varvec{h}}_{N} } \right\} \supseteq , \cdots , \supseteq v_{1} = \left\{ {{\varvec{u}}_{0} ,{\varvec{u}}_{0} + {\varvec{h}}_{1} } \right\} \supseteq v_{0} = \left\{ {{\varvec{u}}_{0} } \right\}, $$

and the corresponding sets of random variables as

3 $$ V_{0} = \left\{ {Z_{0} } \right\} \subseteq V_{1} = \left\{ {Z_{0} ,Z_{1} } \right\} \subseteq , \cdots , \subseteq V_{N} = \left\{ {Z_{0} ,Z_{1} , \ldots , Z_{N} } \right\}. $$

These spatial templates consist of the possible spatial configurations of the partially matched replicates considered in this paper, and the entire set is denoted as $G = \cup_{i = 0}^{N} v_{i}$.

Let the training data from the replicates associated with the $G$ be denoted as ${\mathcal{G}}$. In this paper, only replicates with spatial templates that satisfy Eqs. (2) and (3) are considered, to simplify the implementation of the proposed method. A more general derivation can be found in the Appendix. For any spatial template $v \in G$, the set of random variables associated with $v$ is denoted as $V$ and the replicates corresponding to the spatial template $v$ are noted as ${\mathcal{G}}_{v}$. The size of the set ${\mathcal{G}}_{v}$ is noted as $\left| {{\mathcal{G}}_{v} } \right|$ representing the number of replicates associated with the spatial template $v$. And let the total number of replicates associated with G be $\left| {\mathcal{G}} \right|$. An arbitrary element ${\varvec{\zeta}}_{t,v} \in {\mathcal{G}}_{v}$ represents a sequence of attribute values as

4 $$ {\varvec{\zeta}}_{t,v} = \left\{ {\zeta_{t,i} :i \in v} \right\}, $$

where $\zeta_{t,i}$ are the values from the replicate at the location of node $i$ in the spatial template $v$ and $1 \le t \le \left| {{\mathcal{G}}_{v} } \right|$ corresponds to one of the replicates. The element mapped to the corresponding kernel subspace from ${\varvec{\zeta}}_{v}$ can be represented as

5 $$ \kappa \left[ {{\varvec{\zeta}}_{t,v} } \right] = K_{V} \left( {{\varvec{\zeta}}_{t,v} , \cdot } \right), $$

which is a function element in the kernel space. With the replicates in ${\mathcal{G}}_{v}$ mapping to the kernel space with kernel ${K}_{V}$, the empirical kernel mean $\kappa [{\mathcal{G}}_{v}]$ can be defined as

6 $$\kappa \left[{\mathcal{G}}_{v}\right]=\frac{1}{\left|{\mathcal{G}}_{v}\right|}{\sum }_{t=1}^{|{\mathcal{G}}_{v}|}\kappa \left[{{\varvec{\zeta}}}_{t,v}\right]=\frac{1}{\left|{\mathcal{G}}_{v}\right|}{\sum }_{t=1}^{|{\mathcal{G}}_{v}|}{K}_{V}\left({{\varvec{\zeta}}}_{t,v}, \cdot \right).$$

For any two nodes $v,{v}^{\prime}\in G$ and ${v}^{\prime}\supseteq v$, there would be a hereditary subset of replicates that are generated from the projection of $v^{\prime}$ onto $v$ by restricting the training data ${\mathcal{G}}_{v^{\prime}}$ to the spatial template $v$, and denote this hereditary subset as ${\mathcal{G}}_{v^{\prime}|v}$. Obviously, ${\mathcal{G}}_{v^{\prime}|v}={\mathcal{G}}_{v}$ if ${v}^{\prime}=v$. Given that ${v}^{\prime}\supseteq v$, the projected elements in the original data space, their mapped elements in the kernel spaces and the kernel statistics can be defined similarly as

7 $$ {\varvec{\zeta}}_{{t,v^{\prime}|v}} = \left\{ {\zeta_{t,i} :i \in v^{\prime}|v, 1 \le t \le \left| {{\mathcal{G}}_{{v^{\prime}}} } \right|} \right\}, $$

8 $$ \kappa \left[ {{\varvec{\zeta}}_{{t,v^{\prime}|v}} } \right] = K_{V} \left( {{\varvec{\zeta}}_{{t,v^{\prime}|v}} , \cdot } \right), $$

9 $$ \kappa \left[ {{\mathcal{G}}_{v^{\prime}|v} } \right] = \frac{1}{{\left| {{\mathcal{G}}_{v^{\prime}} } \right|}}\mathop \sum \limits_{t = 1}^{{\left| {{\mathcal{G}}_{{v^{\prime}}} } \right|}} \kappa \left[ {{\varvec{\zeta}}_{{t,v^{\prime}|v}} } \right] = \frac{1}{{\left| {{\mathcal{G}}_{v^{\prime}} } \right|}}\mathop \sum \limits_{t = 1}^{{\left| {{\mathcal{G}}_{{v^{\prime}}} } \right|}} K_{V} \left( {{\varvec{\zeta}}_{{t,v^{\prime}|v}} , \cdot } \right). $$

For convenience of notation, $\kappa \left[ \cdot \right]$ generally represents an element in the kernel space with certain kernel function $K$ that is mapped from the original data space. For instance, $\kappa \left[ {{\varvec{\zeta}}_{{t,v^{\prime}|v}} } \right]$ in Eq. (8) appears as an element embedded in the kernel space from a single replicate ${\varvec{\zeta}}_{{t,v^{\prime}|v}}$, and $\kappa \left[ {{\mathcal{G}}_{v^{\prime}|v} } \right]$ is the sample average of a group of elements embedded into the kernel space from a set of samples ${\mathcal{G}}_{v^{\prime}|v}$. As the kernel space is also a vector space, the kernel statistics $\kappa \left[ {{\mathcal{G}}_{v^{\prime}|v} } \right]$ also lies in the same kernel space as an element.

Then, the aggregated kernel statistics $\kappa \left[ {\mathcal{G}} \right]$ based on the replicates associated to the ensemble of various spatial templates in $G$ can be defined as

10 $$ \kappa \left[ {\mathcal{G}} \right] = \mathop \sum \limits_{n = 1}^{N} \frac{1}{{\mathop \sum \nolimits_{i = n}^{N} \left| {{\mathcal{G}}_{{v_{i} }} } \right|}} \cdot \left( {\mathop \sum \limits_{i = n}^{N} (\kappa \left[ {{\mathcal{G}}_{{v_{i} |v_{n} }} } \right] - \kappa \left[ {{\mathcal{G}}_{{v_{i} |v_{n - 1} }} } \right])\left| {{\mathcal{G}}_{{v_{i} }} } \right|} \right). $$

Combined with Eq. (9), it can be also written as

11 $$ \kappa \left[ {\mathcal{G}} \right] = \mathop \sum \limits_{n = 1}^{N} \frac{1}{{\mathop \sum \nolimits_{i = n}^{N} \left| {{\mathcal{G}}_{{v_{i} }} } \right|}} \cdot \left( {\mathop \sum \limits_{i = n}^{N} \mathop \sum \limits_{t = 1}^{{\left| {{\mathcal{G}}_{{v_{i} }} } \right|}} K_{{V_{n} }} \left( {{\varvec{\zeta}}_{{t,v_{i} |v_{n} }} , \cdot } \right) - K_{{V_{n - 1} }} \left( {{\varvec{\zeta}}_{{t,v_{i} |v_{n - 1} }} , \cdot } \right)} \right). $$

### Sequential Simulation via Statistical Learning with Aggregated Kernel Statistics

The general concept of statistical learning refers to learning any functional dependency from a certain dataset without prior knowledge of the data (Vapnik 1995, 1998). Herein, the statistical learning framework for the high-order sequential simulation, specifically, means to learn the conditional probability distribution based on the observed replicates from the sample data. The learning procedure can be achieved conveniently through an optimization algorithm in the SLM-kernel space. In fact, the kernel mean defines a feature map to embed probability distribution to the associated kernel space (Song et al. 2009; Smola et al. 2007; Muandet et al. 2016). The empirical mean in the kernel space embeds the empirical probability distribution. Similarly, the expectational mean in the kernel space given a certain probability distribution embeds the distribution as an element in the kernel space. Minimizing the distance between the two above-mentioned elements in the kernel space leads to matching of high-order spatial statistics of the target distribution to those of the available data with the kernel space defined by the SLM-kernel.

Equation (11) defines a feature map through the aggregated kernel statistics from an ensemble of kernel subspaces. Suppose that the conditioning data are ${\Lambda } = \left\{ {\zeta_{1} , \ldots ,\zeta_{N} } \right\}$, and define the conditioned kernel statistics $\kappa \left[ {{\mathcal{G}};{\Lambda }} \right]$ as

12 $$\kappa \left[\mathcal{G};\Lambda \right]=\mathop{\sum }\limits_{n=1}^{N}\frac{1}{{\sum }_{i=n}^{N}\left|{\mathcal{G}}_{{v}_{i}}\right|}\cdot \left(\mathop{\sum }\limits_{i=n}^{N}\mathop{\sum }\limits_{t=1}^{\left|{\mathcal{G}}_{{v}_{i}}\right|}{K}_{{V}_{n}}\left({{\varvec{\zeta}}}_{t,{v}_{i}|{v}_{n}},\Lambda \right)-{K}_{{V}_{n-1}}\left({{\varvec{\zeta}}}_{t,{v}_{i}|{v}_{n-1}},\Lambda \right)\right).$$

Furthermore, marginalization of $\kappa \left[\mathcal{G};\Lambda \right]$ can be defined as

13 $$\kappa \left[\mathcal{G}|\Lambda \right]=\frac{\kappa \left[\mathcal{G};\Lambda \right]}{{\int }_{[-\mathrm{1,1}]}\kappa \left[\mathcal{G};\Lambda \right]d{z}_{0}}.$$

The emphasis herein, is to derive a feasible computational model for the marginalized kernel statistics, $\kappa \left[\mathcal{G}|\Lambda \right]$, defined in Eq. (13). An interesting property of SLM-kernel from its definition is

14 $${K}_{V}={{K}_{V\backslash U}K}_{U}$$

where $V\backslash U$ is the set difference between the set of random variables $V$ and $U$ with $V\supseteq U$. It means the high-order dimensional kernels could be built incrementally from the lower-dimensional ones as

15 $${K}_{V}={\prod }_{i=1}^{n}{K}_{{U}_{i}},$$

where ${U}_{i}$ are disjoint subsets of $V$ and $V={\cup }_{i=1}^{n}{U}_{i}$.

Obviously, ${V}_{0}=\{{Z}_{0}\}$ is a single element set and the kernel ${K}_{{V}_{0}}$ can be written as

16 $${K}_{{V}_{0}}\left({z}_{0},{z}_{0}^{\prime}\right)=\mathop{\sum }\limits_{w=0}^{W}\left(w+\frac{1}{2}\right){P}_{w}\left({z}_{0}\right){P}_{w}\left({z}_{0}^{\prime}\right).$$

Noting the orthogonal property of Legendre polynomials, it is easy to derive that

17 $${\int }_{\left[-\mathrm{1,1}\right]}{K}_{{V}_{0}}\left({\zeta }_{t,0}, {z}_{0}\right)d{z}_{0}=1,$$

and therefore, there is

18 $${\int }_{\left[-\mathrm{1,1}\right]}\kappa \left[\mathcal{G};\Lambda \right]d{z}_{0}=\mathop{\sum }\limits_{n=1}^{N}\frac{1}{{\sum }_{i=n}^{N}\left|{\mathcal{G}}_{{v}_{i}}\right|}\cdot \left(\mathop{\sum }\limits_{i=n}^{N}\mathop{\sum }\limits_{t=1}^{\left|{\mathcal{G}}_{{v}_{i}}\right|}{K}_{{V}_{n}\backslash {V}_{0}}\left({{\varvec{\zeta}}}_{t,{v}_{i}|{v}_{n}},\Lambda \right)-{K}_{{V}_{n-1}\backslash {V}_{0}}\left({{\varvec{\zeta}}}_{t,{v}_{i}|{v}_{n-1}},\Lambda \right)\right)$$

According to Eq. (16), the result of Eq. (19) can be obtained from the intermediate result of computing Eq. (12). In the end, $\kappa \left[\mathcal{G}|\Lambda \right]$ can be expressed in the form as

19 $$\kappa \left[\mathcal{G}|\Lambda \right]=\mathop{\sum }\limits_{t=1}^{\left|\mathcal{G}\right|}{\beta }_{t}{K}_{{V}_{0}}\left({\zeta }_{t,0},{z}_{0}\right),$$

where ${\beta }_{t}$ are constant coefficients that can be computed through Eqs. (13) and (18). Equation (19) is a linear combination of elements in kernel space determined by kernel ${K}_{{V}_{0}}$, and therefore marginalization of the aggregated kernel statistics, $\kappa \left[\mathcal{G}|\Lambda \right]$, embeds the empirical conditional probability distribution to the corresponding kernel space with kernel ${K}_{{V}_{0}}$. In other words, $\kappa \left[\mathcal{G}|\Lambda \right]$ is an element in the kernel space containing the high-order spatial information from the replicates found in the sample data given the conditioning data as $\Lambda$. The purpose of the proposed method is to find a target distribution $\widehat{p}$ as the CPDF at each node encountered in the sequential simulation procedure. The expected kernel statistics with distribution $\widehat{p}$ can be defined as

20 $$\kappa \left[\widehat{p}\right]={E}_{{z}_{0}^{\prime}\sim \widehat{p}}\left[\phi \left({z}_{0}^{\prime}\right)\right]={E}_{{z}_{0}^{\prime}\sim \widehat{p}}\left[{K}_{{V}_{0}}\left({z}_{0}^{\prime},{z}_{0}\right)\right],$$

where $\phi \left({z}_{0}^{\prime}\right)={K}_{{V}_{0}}\left({z}_{0}^{\prime},{z}_{0}\right)$ defines the feature mapping function in the kernel space associated to kernel ${K}_{{V}_{0}}$, and ${E}_{{z}_{0}^{\prime}\sim \widehat{p}}[\phi \left({z}_{0}^{\prime}\right)]$ means the expectation of the features mapped from elements in the original data space with a probability distribution $\widehat{p}$. Thus, the two elements embedding into the kernel space $\mathcal{H}$ associated to kernel ${K}_{{V}_{0}}$ are represented as $\kappa \left[\mathcal{G}|\Lambda \right]$ and $\kappa \left[\widehat{p}\right]$, corresponding to the replicates from the sample data and the target CPDF in the simulation, respectively. Given a convex space ${\mathcal{P}}_{0}$ as the solution space of this target distribution $\widehat{p}$, minimizing the difference between these two elements $\kappa \left[\mathcal{G}|\Lambda \right]$ and $\kappa \left[\widehat{p}\right]$ results in a target CPDF that matches the high-order spatial statistics of the replicates from the sample data. Therefore, the target CPDF $\widehat{p}$ can be solved by the below minimization problem as

21 $$\underset{\widehat{p}}{\mathrm{min}}{{\Vert \kappa \left[\mathcal{G}|\Lambda \right]-\kappa \left[\widehat{p}\right]\Vert }^{2}}_{\mathcal{H}}.$$

The minimization in Eq. (21) can be expanded to a quadratic programming problem by noticing that the inner products can be expressed as kernel functions. The details to solve the problem given $\widehat{p}$ as a convex combination of certain prototype distributions is established in Yao et al. (2020) and thus will not be repeated here. It should be noted that although Eq. (19) appears in a similar form as Eq. (16) in Yao et al. (2020), the coefficients ${\beta }_{t}$ in Eq. (19) depend on the aggregated kernel statistics with different spatial templates, which is critical for the utilization of information from partially matched replicates.

**Fig. 1:**
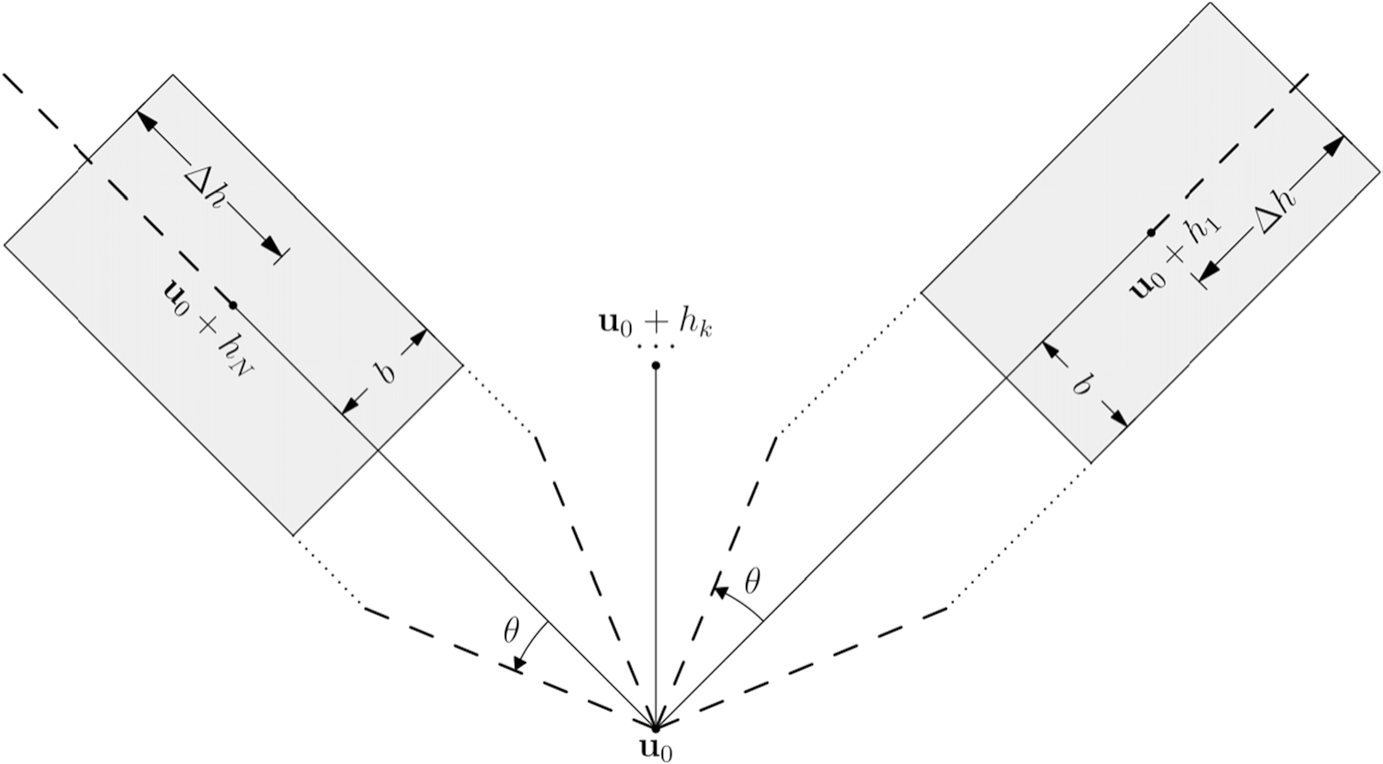
Tolerances along each distance vector of the spatial template for retrieving replicates from the samples

With the computation of aggregated kernel statistics of various spatial templates and the auxiliary procedure to estimate the conditional probability distribution, the sequential simulation method via statistical learning with aggregated kernel statistics can be described as the following:

1. Transform the sample data to the interval [− 1, 1] of Legendre polynomials.
2. Initialize a random path to visit the simulation grid.
3. For each node to be simulated, find the conditioning data as the data event. The nodes from the spatial template of the data event are ordered increasingly from their distances to the center node.
4. For each distance vector in the spatial template, allow certain angle tolerance $\theta$ and lag tolerance $\Delta h$ as well as a bandwidth $b$ to find matched node from the samples (Fig. 1). Start from the distance vector nearest to the center node and go through all the distance vectors orderly until no matching node is found from the samples. Scan the entire sample dataset and store the replicates to separate lists according to the number of nodes matched to the spatial template of the data event.
5. Compute the aggregated kernel statistics from the partially matched replicates retrieved in Step (4) following Eqs. (12) and (18).
6. Compute the marginalized kernel statistics defined by Eq. (13) and the feature map $\kappa \left[\mathcal{G}|\Lambda \right]$ defined by Eq. (19), solve the minimization problem in Eq. (21) to get an estimated conditional probability distribution. Draw a random sample from the estimated probability distribution and add the value to the simulation grid.
7. Repeat from step (3) until all the nodes of the simulation grid are visited.
8. Back transform the simulate grid from the interval [− 1, 1] to generate a realization in the original data space.

## Case Study with a Synthetic Dataset

**Fig. 2:**
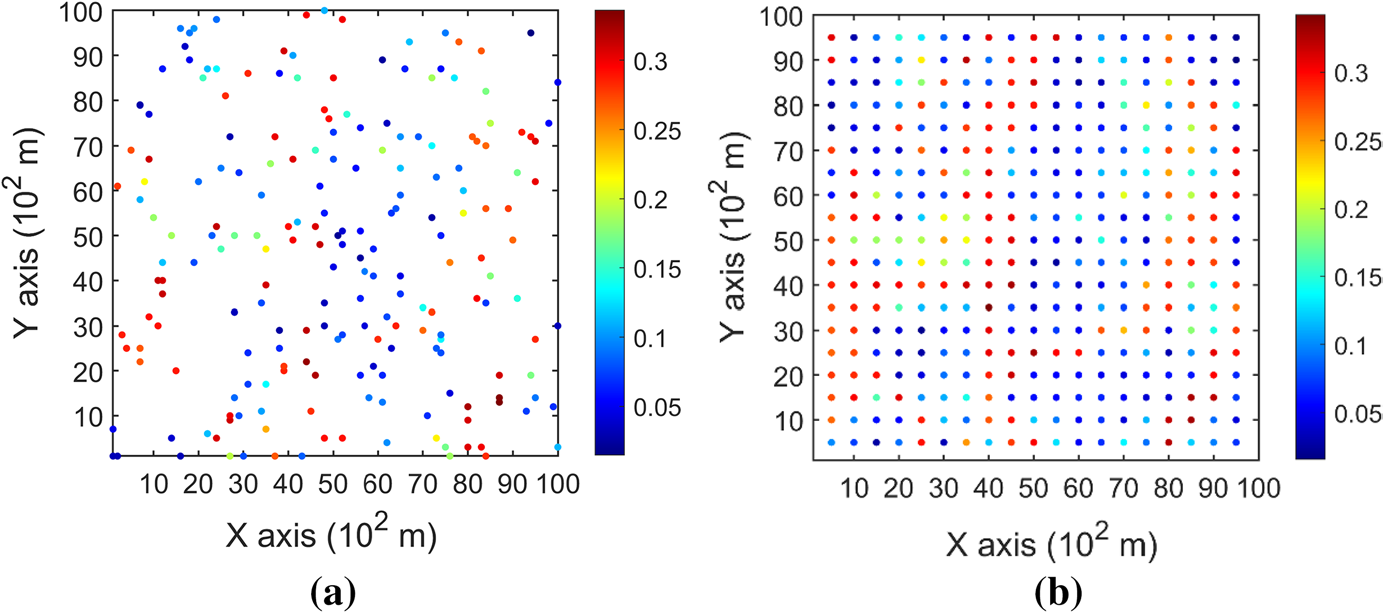
Two different sample datasets. **a** DS-1 with 200 randomly drawn samples, **b** DS-2 with 400 samples

**Fig. 3:**
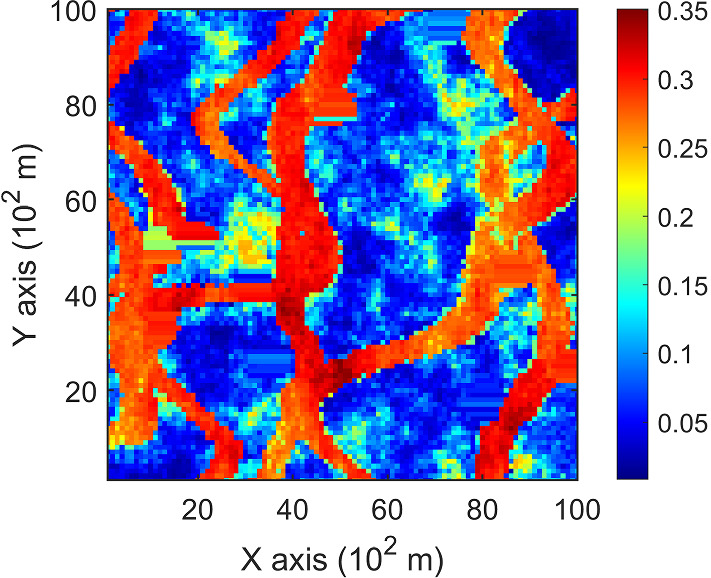
A horizontal section of porosity attribute from a reservoir, acting as the exhaustive image

The synthetic data are a horizontal section extracted from a fully known reservoir dataset of porosity (Mao and Journel 1999). Two different sample datasets are drawn from the section representing different sampling density. The dataset DS-1 contains samples randomly drawn from 200 locations, and the dataset DS-2 has 400 samples with regular spacing. Figure 2 shows the samples, and Fig. 3 displays the exhaustive image.

**Fig. 4:**
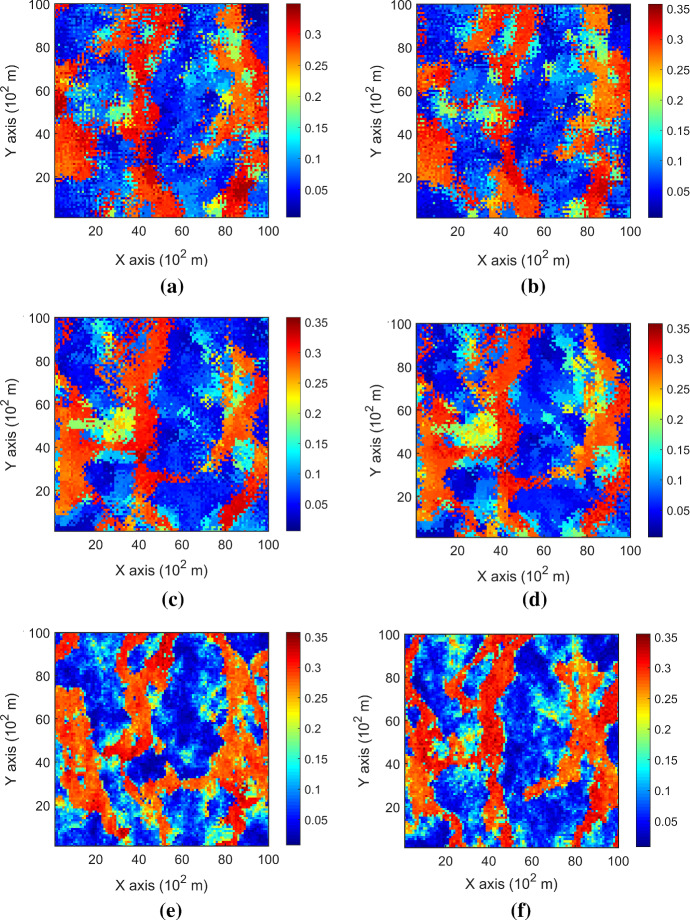
Realizations of TI-free high-order simulation with the sample data DS-1 in **a**, **b** and with the sample data DS-2 in **c, d**; for comparison, realizations of high-order simulation using a TI with the sample data DS-1 in **e** and with the sample data DS-2 in **f** (from Yao et al. 2020)

Two realizations of the proposed high-order simulation method using DS-1 and DS-2 are demonstrated in Fig. 4a–d, respectively. The same random paths are used for the two realizations for comparison of the impact of sampling density on the simulation method. The visual comparison with the exhaustive image shows that both realizations reproduce the preferential channels along the vertical direction. This shows that the proposed method has the generalization capacity to provide stability of simulation with relatively sparse data. On the other hand, the realizations using DS-2 as the sample data retain more fine structures as well as the overall spatial connectivity than the other realization. The reason is that a sparser dataset in general has fewer replicates for small structures and, thus, the estimated high-order spatial statistics have to be generalized to stabilize the statistical inference in the situation that the replicates are fewer. Generally speaking, as the amount of data increases, the models tend to have more variations in finer spatial structures and vice versa.

**Fig. 5:**
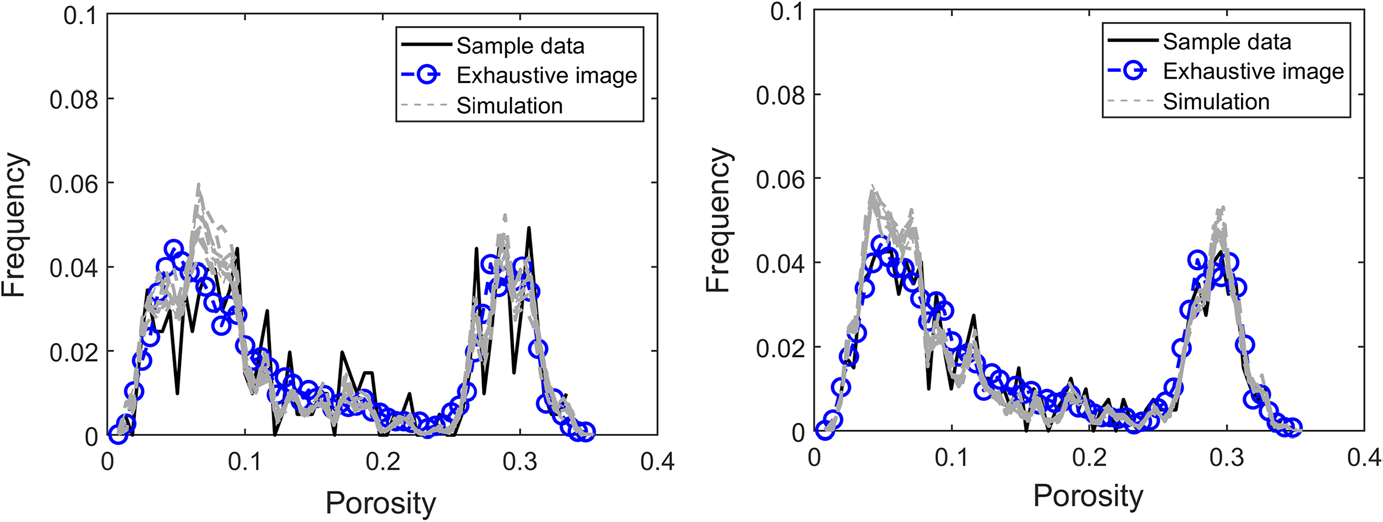
Histograms of the sample data, the exhaustive image, and ten realizations using a DS-1 and b DS-2 as the sample data, respectively

To further demonstrate the TI-free feature of the proposed simulation method, two realizations of the high-order simulation based on statistical learning using a TI from Yao et al. (2020) are displayed in Fig. 4e, f, for comparison. The results show that the TI adds complementary information to finer structures of the realizations. As the samples are relatively dense, the contribution of the additional information from the TI also becomes less important since the TI-free simulation method can generate more details from the available sample data. The comparison of histograms of ten realizations with DS-1 and DS-2 as the sample data with the histograms of the two sample datasets, as well as the exhaustive image, is demonstrated in Fig. 5. In both cases, the histograms of the realizations follow the histograms of the sample datasets, whereas the one with dense data resembles more the exhaustive image, as expected.

**Fig. 6:**
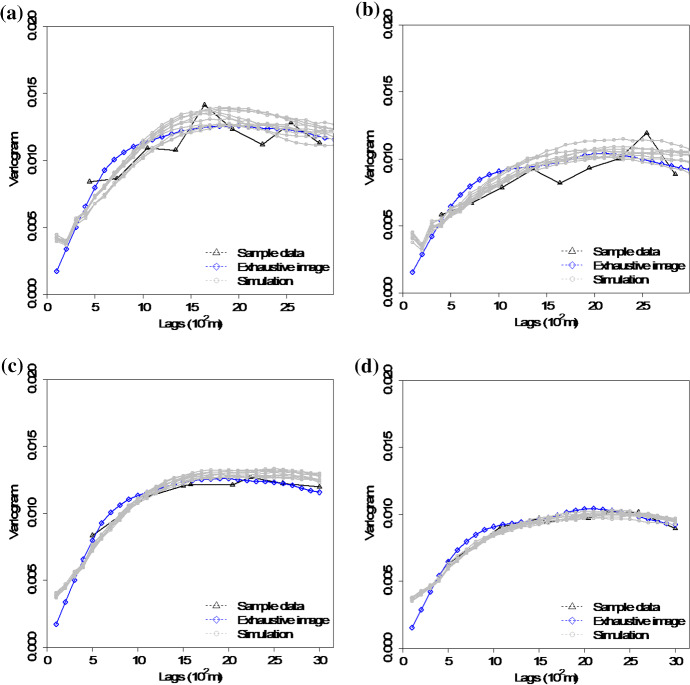
Variograms of ten realizations. **a**, **b** Along *X* and *Y* axis with DS-1 as the sample data; **c**, **d** along *X* and *Y* axis with DS-2 as the sample data.

**Fig. 7:**
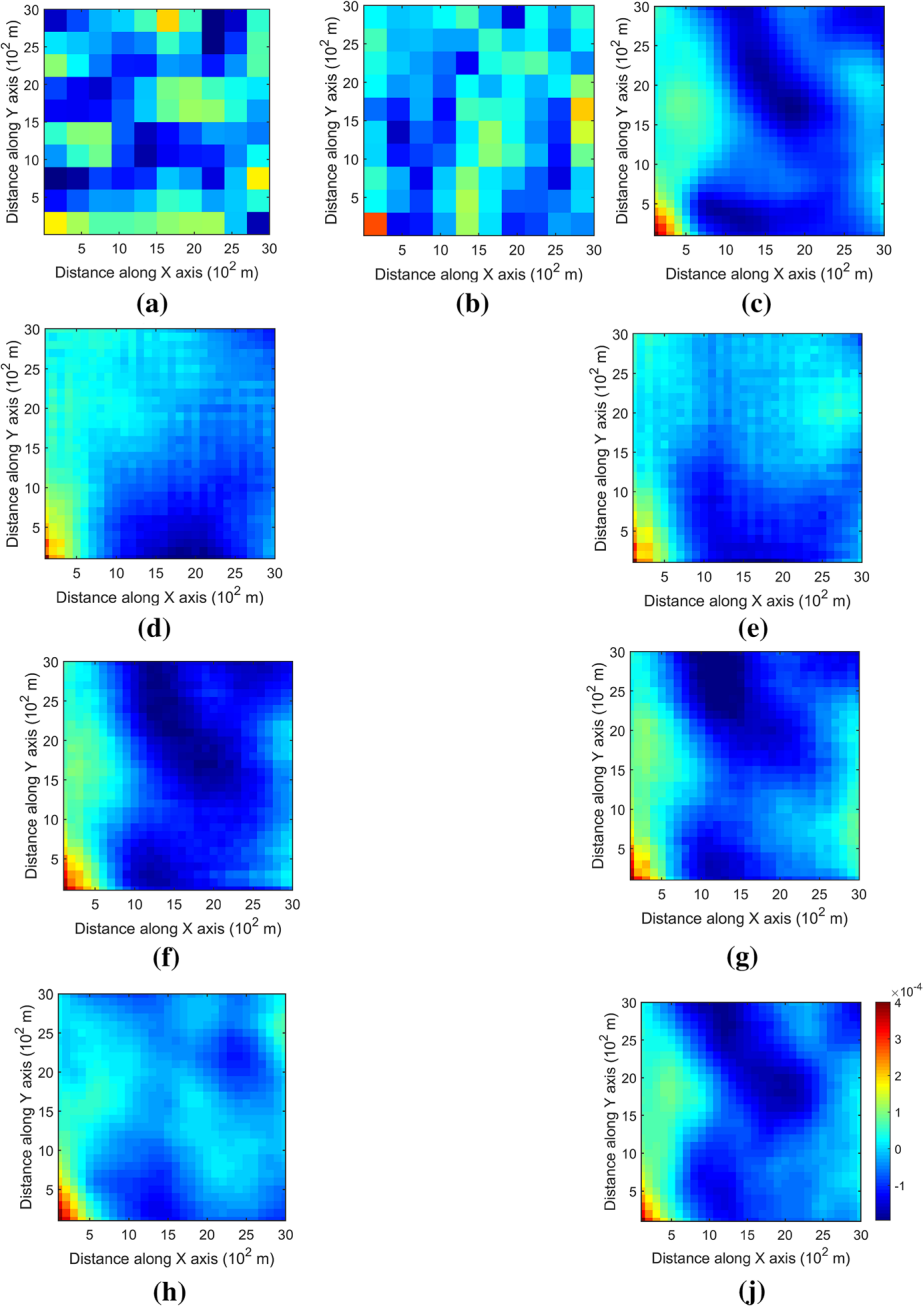
Third-order cumulant maps of **a** DS-1, **b** DS-2, **c** exhaustive image, **d**, **e** realizations in Fig. 4a, b with DS-1 as the sample data, **f**, **g** realizations in Fig. 4c, d with DS-2 as the sample data; **h**, **i** realizations of high-order simulation using a TI with DS-1 and DS-2 as the sample data, respectively (from Yao et al. (2020))

**Fig. 8:**
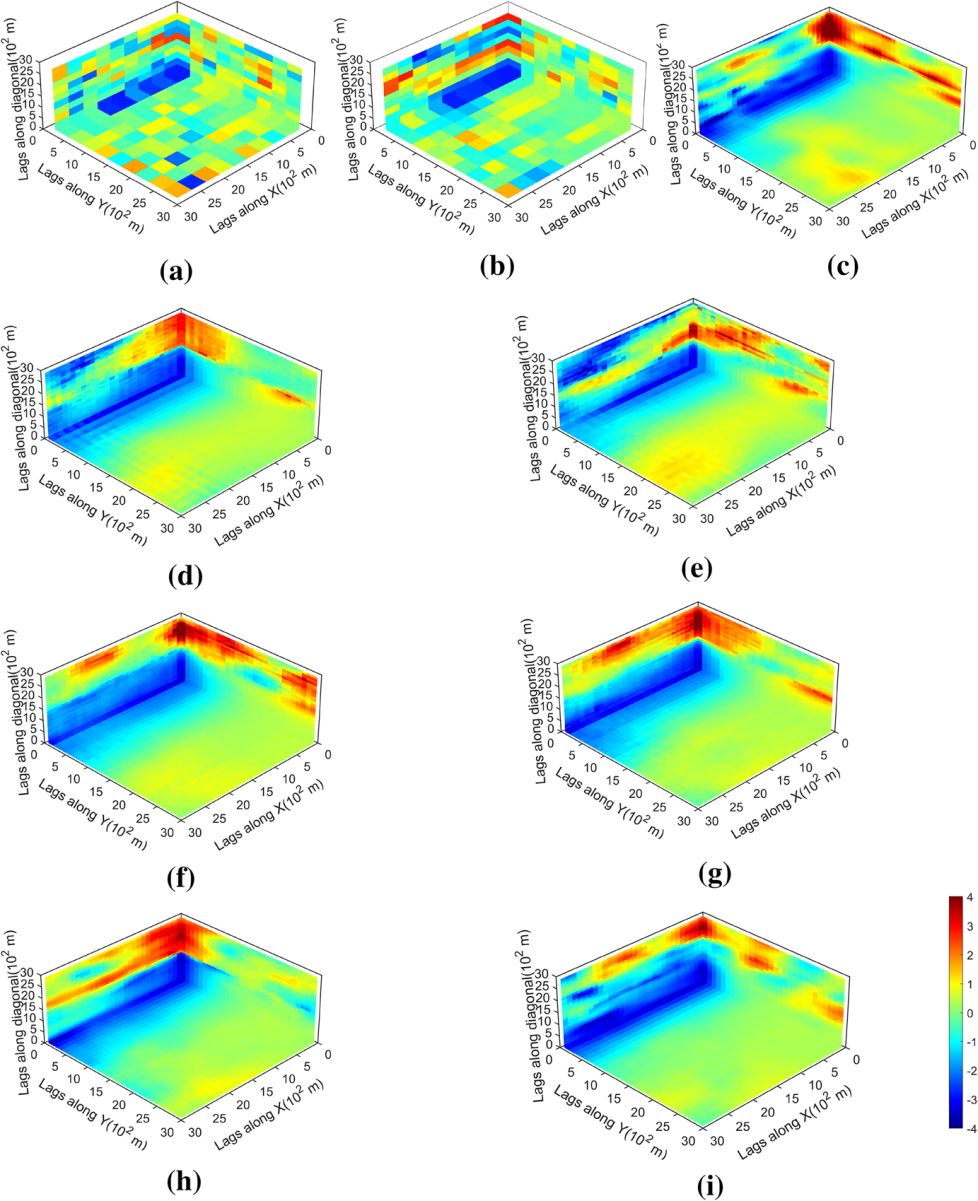
Fourth-order cumulant maps of **a** DS-1, **b** DS-2, **c** exhaustive image; **d**, **e** realizations in Fig. 4a, b with DS-1 as the sample data; **f**, **g** realizations in Fig. 4c, d with DS-2 as the sample data; **h**, **i** realizations of high-order simulation using a TI with DS-1 and DS-2 as the sample data, respectively (from Yao et al. 2020)

The variograms of ten realizations based on the proposed simulation method using the two different sample datasets are shown in Fig. 6, showing that the simulations reproduce the variograms of the samples. The third-order cumulant maps of the sample data and the corresponding realizations with the proposed simulation method are shown in Fig. 7. Furthermore, the fourth-order cumulate maps of the sample data and the realizations are displayed in Fig. 8 for comparison. In this example, the third-order cumulant maps are calculated based on a spatial template along *X* and *Y* axes with varied lengths in both directions. The spatial templates of the fourth-order cumulants include extra distance vectors along the diagonal direction in addition to the two axes directions. The fourth-order cumulant maps are also scaled by their deviations for better contrast of the patterns. In general, these high-order cumulant maps represent more complex spatial patterns that characterize interrelations among multiple points. The cumulant maps of two representative realizations from the high-order simulation based on statistical learning using a TI are displayed in the bottom of Figs. 7 and 8 for comparison with the results from the proposed method. The comparisons of the cumulant maps suggest that the proposed method is able to reproduce the high-order spatial statistics of the sample data as well as the exhaustive image. The results above show that the proposed approach leads to a reliable inference on the underlying random field model, given a reasonable number of samples available, and thus avoids the potential statistical conflicts using a TI to carry out the high-order simulation.

## Conclusions

This paper presents a high-order sequential simulation approach based on statistical learning with aggregated kernel statistics from a set of sample data. Regarding the sparsity of the sample data used to infer the high-order spatial statistics of the underlying random field model, the partially matched replicates of the data events encountered in the simulation are mapped into kernel subspaces. The latter kernel subspaces are defined by different kernel functions corresponding to different configurations of spatial templates to create an ensemble set of elements in kernel subspaces. The ensemble of elements in the kernel subspaces are aggregated to construct the new concept of aggregated kernel statistics. The aggregated kernel statistics are crucial in building a new feature map to consider partially matched replicates together in the same kernel space of the conditional probability distribution. In addition, the statistical learning framework for high-order simulation offers generalization capacity for sparse data learning. The combination of the aggregated kernel statistics with the statistical learning thus provides a new way to derive the proposed TI-free high-order simulation method. The proposed method tackles the issue of statistical conflicts between the sample data and the TI. The case study from the fully known dataset shows that the proposed method reproduces both lower-order and higher-order spatial statistics in generated realizations. Even with relatively sparse samples, the proposed method retains the main spatial patterns of the available data, which is characterized by high-order spatial statistics. However, the simulation results of the proposed method generally exhibit higher discontinuity in the short range than the simulation results using a TI. In contrast to the variograms in the second-order geostatistical simulation methods, the high-order spatial statistics are taken into account through a statistical learning process. This is also different from the B-Spline model to fit the high-order spatial moments of categorical data developed in Minniakhmetov and Dimitrakopoulos (2017). Specifically, the boundary conditions that are important to build the B-Spline model in Minniakhmetov and Dimitrakopoulos (2017) cannot apply to continuous data. The high-order spatial statistics of the random field are rather equipped by implicit modeling from the learning algorithm in the current approach, while the accuracy of modeling is data dependent. A possible strategy for further improvement of the results could be utilization of short-range high-order spatial information from other complementary sources. It should be noted that the concept of aggregated kernel statistics is quite flexible and can accommodate information from different data sources with various spatial configurations. This represents a potential direction for future research.

## Figures and Tables

**Fig. 9:**
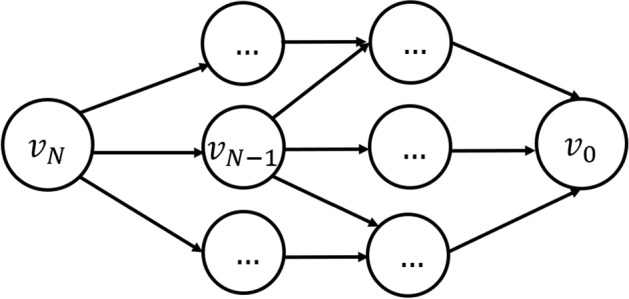
Sketch graph of relations between subsets of a certain spatial template

## Appendix: Derivation of Aggregated Kernel Statistics

The more general relations among the replicates associated to various spatial templates can be analogous to a flow network $G$ with a source corresponding to the full spatial template ${v}_{N}$ and a sink corresponding to a single center node ${v}_{0}$. The arrows represent the subset relation between two spatial templates. In general, the replicates associated with a certain spatial template $v\in G$ receive inflows from the ancestor nodes corresponding to the subsets of replicates projected on $v$ from those replicates with a larger size template. At the same time, the replicates associated with a certain spatial template $v\in G$ also has outflows to their direct children nodes, which can be represented as ${O}_{v}=\{{v}^{\prime\prime}\subset v,\nexists r\in G s.t. {v}^{\prime\prime}\subset r\subset v\}$. The idea of deriving the aggregated kernel statistics is to isolate the computation of kernel statistics with the spatial template $v^{\prime\prime}$ from the current spatial template $v$, while augmenting the kernel statistics from its ancestor templates. Thus, for the ensemble replicates $\mathcal{G}$ with spatial templates in $G$, the aggregated kernel statistics are computed as

22 $$ \kappa \left[\mathcal{G}\right]=\mathop{\sum }\limits_{v\in G}\frac{1}{{\sum }_{{v}^{\prime}\supseteq v}\left|{\mathcal{G}}_{{v}^{\prime}}\right|}\mathop{\sum }\limits_{\begin{array}{c}{v}^{\prime}\supseteq v\\ {v}^{\prime\prime}\in {O}_{v}\end{array}}\left(\kappa \left[{\mathcal{G}}_{{v}^{\prime}|v}\right]-\kappa \left[{\mathcal{G}}_{v|{v}^{\prime\prime}}\right]\right)\cdot \left|{\mathcal{G}}_{{v}^{\prime}}\right| .$$

The computation will have to traverse all the nodes in graph $G$. A formal proof of Eq. (22) originates from the equivalency of SLM-kernel statistics and the computational model of probability density approximation based on spatial Legendre moments, as detailed in Yao et al. (2018, 2020). For a certain spatial template $v$ of size (n + 1) and the corresponding replicates as ${\mathcal{G}}_{v}$, the kernel statistics defined in Eq. (6) are equivalent to

23 $$\kappa \left[{\mathcal{G}}_{v}\right]={\sum }_{{w}_{0}=0}^{W}{\sum }_{{w}_{1}=0}^{W}\cdots {\sum }_{{w}_{n}=0}^{W}{L}_{{w}_{0}{w}_{1}\cdots {w}_{n}}{\prod }_{i=0}^{n}{P}_{{w}_{i}}\left({z}_{i}\right),$$

where $W$ is the maximal degree of polynomial series and ${P}_{{w}_{i}}$ is the Legendre polynomial of order ${w}_{i}$. ${L}_{{w}_{0}{w}_{1}\cdots {w}_{n}}$ is the spatial Legendre moment and can be numerically approximated as (Yao et al. 2018)

24 $${L}_{{w}_{0}{w}_{1}\cdots {w}_{n}}\approx \frac{1}{\left|{\mathcal{G}}_{v}\right|}{\sum }_{t=1}^{\left|{\mathcal{G}}_{v}\right|}{\prod }_{i=0}^{n}{P}_{{w}_{i}}\left({\zeta }_{t,i}\right),$$

where ${\zeta }_{t,i}$ means the attribute value of node $i$ in $t$-th replicate from ${\mathcal{G}}_{v}$. Considering another spatial template ${v}^{\prime}$ of size $({n}^{\prime}+1)$ with ${v}^{\prime}\supseteq v$ and ${n}^{\prime}>n$, one can see that

25 $${L}_{{w}_{0}{w}_{1}\cdots {w}_{n}}\approx \frac{1}{\left|{\mathcal{G}}_{v}\right|+\left|{\mathcal{G}}_{{v}^{\prime}}\right|}\left[{\sum }_{t=1}^{\left|{\mathcal{G}}_{v}\right|}{\prod }_{i=0}^{n}{P}_{{w}_{i}}\left({\zeta }_{t,i}\right)+{\sum }_{t=1}^{\left|{\mathcal{G}}_{{v}^{\prime}}\right|}{\prod }_{i=0}^{n}{P}_{{w}_{i}}\left({\zeta }_{t,i}\right)\right].$$

On the other hand, any spatial Legendre moments containing nodes $n<{m}_{j}\le n^{\prime}(1\le j\le {n}^{\prime}-n)$ can only be computed from the replicates ${\mathcal{G}}_{{v}^{\prime}}$ but excluding ${\mathcal{G}}_{v}$. Note Eq. (23) that these computations can be implemented by the difference of kernel statistics corresponding to the spatial templates $v^{\prime}$ and $v$, and the generalization to Eq. (22) is straightforward. The current paper considers one branch as Eq. (2) implies. And with this assumption, Eq. (10) can be easily derived from Eq. (22) (Fig. 9).
